# Supplementary material for: Sero-prevalence and associated factors of sexually transmitted infections among youth-friendly services Attendees
Source: PLoS One. 2023 Jan 23;18(1):e0279900. doi: 10.1371/journal.pone.0279900 (PMC9870153; doi:10.1371/journal.pone.0279900)
Supplement: Attachment — Submitted filename: Response to reviewers comments.docx [file pone.0279900.s002.docx]

PONE-D-22-12316

Sero-prevalence and associated factors of sexually transmitted infections among youth-friendly services attendants

PLOS ONE

Dear Dr. Ali,

Thank you for submitting your manuscript to PLOS ONE. After careful consideration, we feel that it has merit but does not fully meet PLOS ONE’s publication criteria as it currently stands. Therefore, we invite you to submit a revised version of the manuscript that addresses the points raised during the review process.

Your manuscript has been assessed by two peer-reviewers and their comments are appended below.

The reviewers comment that your manuscript requires further information and/or clarification on the methodology and statistics used in this study. In addition, the reviewers have raised major concerns with the presentation of the results and discussion of this study. Furthermore, the reviewers have commented that your manuscript does not adequately discuss the limitations to this study.

Could you please revise the manuscript to carefully address the concerns raised?

Response: We have included the limitation suggested.

Please submit your revised manuscript by Nov 09 2022 11:59PM. If you will need more time than this to complete your revisions, please reply to this message or contact the journal office at plosone@plos.org. When you're ready to submit your revision, log on to https://www.editorialmanager.com/pone/ and select the 'Submissions Needing Revision' folder to locate your manuscript file.

Please include the following items when submitting your revised manuscript:

A rebuttal letter that responds to each point raised by the academic editor and reviewer(s). You should upload this letter as a separate file labeled 'Response to Reviewers'.

A marked-up copy of your manuscript that highlights changes made to the original version. You should upload this as a separate file labeled 'Revised Manuscript with Track Changes'.

An unmarked version of your revised paper without tracked changes. You should upload this as a separate file labeled 'Manuscript'.

If you would like to make changes to your financial disclosure, please include your updated statement in your cover letter. Guidelines for resubmitting your figure files are available below the reviewer comments at the end of this letter.

If applicable, we recommend that you deposit your laboratory protocols in protocols.io to enhance the reproducibility of your results. Protocols.io assigns your protocol its own identifier (DOI) so that it can be cited independently in the future. For instructions see: https://journals.plos.org/plosone/s/submission-guidelines#loc-laboratory-protocols. Additionally, PLOS ONE offers an option for publishing peer-reviewed Lab Protocol articles, which describe protocols hosted on protocols.io. Read more information on sharing protocols at https://plos.org/protocols?utm_medium=editorial-email&utm_source=authorletters&utm_campaign=protocols.

We look forward to receiving your revised manuscript.

Kind regards,

Maria Elisabeth Johanna Zalm, Ph.D

Editorial Office

PLOS ONE

Journal requirements:

When submitting your revision, we need you to address these additional requirements.

1.Please ensure that your manuscript meets PLOS ONE's style requirements, including those for file naming. The PLOS ONE style templates can be found at

https://journals.plos.org/plosone/s/file?id=wjVg/PLOSOne_formatting_sample_main_body.pdf and

<https://journals.plos.org/plosone/s/file?id=ba62/PLOSOne_formatting_sample_title_authors_affiliations.pdf>.

Response: OK

2.Thank you for stating the following in your Competing Interests section:

“no competing interests exist.”

Please complete your Competing Interests on the online submission form to state any Competing Interests. If you have no competing interests, please state ""The authors have declared that no competing interests exist."", as detailed online in our guide for authors at http://journals.plos.org/plosone/s/submit-now

This information should be included in your cover letter; we will change the online submission form on your behalf.

Response: We have included as suggested.

3.In your Data Availability statement, you have not specified where the minimal data set underlying the results described in your manuscript can be found. PLOS defines a study's minimal data set as the underlying data used to reach the conclusions drawn in the manuscript and any additional data required to replicate the reported study findings in their entirety. All PLOS journals require that the minimal data set be made fully available. For more information about our data policy, please see <http://journals.plos.org/plosone/s/data-availability>.

Response: We have uploaded raw data used for analysis.

Upon re-submitting your revised manuscript, please upload your study’s minimal underlying data set as either Supporting Information files or to a stable, public repository and include the relevant URLs, DOIs, or accession numbers within your revised cover letter. For a list of acceptable repositories, please see http://journals.plos.org/plosone/s/data-availability#loc-recommended-repositories. Any potentially identifying patient information must be fully anonymized.

Important: If there are ethical or legal restrictions to sharing your data publicly, please explain these restrictions in detail. Please see our guidelines for more information on what we consider unacceptable restrictions to publicly sharing data: http://journals.plos.org/plosone/s/data-availability#loc-unacceptable-data-access-restrictions. Note that it is not acceptable for the authors to be the sole named individuals responsible for ensuring data access.

We will update your Data Availability statement to reflect the information you provide in your cover letter.

4.We note that you have indicated that data from this study are available upon request. PLOS only allows data to be available upon request if there are legal or ethical restrictions on sharing data publicly. For more information on unacceptable data access restrictions, please see http://journals.plos.org/plosone/s/data-availability#loc-unacceptable-data-access-restrictions.

In your revised cover letter, please address the following prompts:

Response: We have uploaded raw data used for analysis.

a) If there are ethical or legal restrictions on sharing a de-identified data set, please explain them in detail (e.g., data contain potentially sensitive information, data are owned by a third-party organization, etc.) and who has imposed them (e.g., an ethics committee). Please also provide contact information for a data access committee, ethics committee, or other institutional body to which data requests may be sent.

b) If there are no restrictions, please upload the minimal anonymized data set necessary to replicate your study findings as either Supporting Information files or to a stable, public repository and provide us with the relevant URLs, DOIs, or accession numbers. For a list of acceptable repositories, please see http://journals.plos.org/plosone/s/data-availability#loc-recommended-repositories.

We will update your Data Availability statement on your behalf to reflect the information you provide.

5.Please upload a copy of Figure 4, to which you refer in your text on page 15. If the figure is no longer to be included as part of the submission please remove all reference to it within the text.

[Note: HTML markup is below. Please do not edit.]

Reviewers' comments:

Reviewer's Responses to Questions

Comments to the Author

1. Is the manuscript technically sound, and do the data support the conclusions?

The manuscript must describe a technically sound piece of scientific research with data that supports the conclusions. Experiments must have been conducted rigorously, with appropriate controls, replication, and sample sizes. The conclusions must be drawn appropriately based on the data presented.

Reviewer #1: Yes

Reviewer #2: Partly

2. Has the statistical analysis been performed appropriately and rigorously?

Reviewer #1: Yes

Reviewer #2: No

3. Have the authors made all data underlying the findings in their manuscript fully available?

The PLOS Data policy requires authors to make all data underlying the findings described in their manuscript fully available without restriction, with rare exception (please refer to the Data Availability Statement in the manuscript PDF file). The data should be provided as part of the manuscript or its supporting information, or deposited to a public repository. For example, in addition to summary statistics, the data points behind means, medians and variance measures should be available. If there are restrictions on publicly sharing data—e.g. participant privacy or use of data from a third party—those must be specified.

Reviewer #1: Yes

Reviewer #2: No

4. Is the manuscript presented in an intelligible fashion and written in standard English?

PLOS ONE does not copyedit accepted manuscripts, so the language in submitted articles must be clear, correct, and unambiguous. Any typographical or grammatical errors should be corrected at revision, so please note any specific errors here.

Reviewer #1: Yes

Reviewer #2: No

5. Review Comments to the Author

Please use the space provided to explain your answers to the questions above. You may also include additional comments for the author, including concerns about dual publication, research ethics, or publication ethics. (Please upload your review as an attachment if it exceeds 20,000 characters)

Reviewer #1: The paper is generally well written and structured. However, in my opinion the paper has some shortcomings in regards to some data analyses and text, and I feel this unique dataset has not been utilized to its full extent. Below I have provided numerous remarks on the text as it is often vague and long-winded. In the abstract please state your recommendation.

1) How can study only 4 microorganisms (HIV, HBV, HCV and T.palladium) represent STI.

**Response:** Thank you for the comment. We did not mean HIV, HBV, HCV, and T. palladium represent STI. We operationally defined STI for this study. Moreover we have mentioned the limitation of operational definition.

2) Why you were use 50% p-value to calculate the sample size? Because there are a lot of study conducted in your country.

**Response:** Yes it is true there several studies which separately addressed STI in non-adolescent population and there are few studies of this kind among the study group. As we have included >2 disease to describe STI we have used a prevalence 50% to represent all of them as it will give maximum sample size.

3) Study about N. gonorrhoeae is you objective? If it is your objective the result obtained must be included into total number of STI. Unless it better to delete.

**Response:** We accept the comment; accordingly have deleted information related to N. gonorrhoeae

4) Please edit your operational definition for STI as “STI is defined as a positive status either for single or combination of infections specific to HIV, HBV, HCV, and syphilis”.

**Response**: We have corrected accordingly as shown in the manuscript with track change.

5) Laboratory test methods you were used are not Gold standard except for the culture. Therefore, what types of quality assurance were conducted to believe your finding?

**Response**: in addition to inbuilt quality control, we used External quality control sample from blood bank and public health institute laboratory to verify test kits.

6) Some Variables in table were not clear and not specific (history of STIs, Recent signs and symptoms of STI). Please show the exact period.

**Response:** We have corrected accordingly for both variables.

7) Include P-value of COR in the table 3.

**Response:** we have included it.

8) To state you result for syphilis to was prefer to use the word “RPR’. What is the reason?

**Response**: We used ‘RPR’ instead of ‘syphilis’ because being reactive for RPR may not indicate active disease ‘syphilis’. To reduce confusion we replaced ‘RPR’ with ‘syphilis’

9) On page 15 line 267 you cite Figure 4. However, there is no any figure in the manuscript.

**Response**: We have removed figure 4 as we have not used it.

10) In Discussion part: page 18 line 288-291. According to you result the CI for the prevalence of STI was 8.7%- 14.9%. Therefore, how can it be lower than study conducted from Jimma (14.3%).

**Response:** We agree with the comment and corrected it as shown in the manuscript with track change.

11) Discussion is good. But it very nice if the sequence is similar with subheading of result ( Socio-demographic, knowledge and behavior, sero-prevalence, risk factor)

**Response:** Thank you for the comment; we have organized the discussion section based on our objectives: seroprevalence of STI and Factors associated with it. We believe socio-demographic and behavioural variables are addressed in the risk factor analysis.

12) In discussion part, most of your justification for your finding does not have references.

**Response**: We have compared the finding of the current study with other studies. We tried to include references wherever they are relevant. We have included our opinion after assessing ours and others that is why some description lacks references.

13) Authors’ contribution: from three authors only two of them were approve this manuscript. Why not all.

**Response:** Thank you for the comment, we have corrected as suggested.

14) instances I also suggested to cite more relevant and recent literature

**Response:** There are scarce similar published articles from Ethiopia. We have tried to include relevant publication as shown in the manuscript with track change. We have added references in the revised manuscript.

15) Correct your reference style

**Response:** we have revised the reference style

Reviewer #2: This an excellent start to an important article that highlights the high STI burden among youth in Ethiopia.

I do have some major concerns, especially pertaining to the presentation of results and discussion.

In general:

Be careful with sweeping statements like at-risk partners (what does this mean?). There are other examples throughout.

**Response:** Thank you for the comment we have tried to remove or correct those phrases as shown in the manuscript with track change.

- the manuscript should be checked for grammar, writing and capitalization of proper nouns.

**Response:** We revised the English as shown in the manuscript with track change.

- The inclusion of swabbing urogenital sites (which ones?) was included briefly in methods and results write up, but does not seem pertinent to seroprevalence and not discussed in the abstract or discussion. Suggest taking this section out or including it fully.

**Response:** As suggested by both reviewers we have removed information related to N. gonorrhoeae

- all acronyms should be defined at first use

**Response:** We have revised as suggested.

General Ethical issues:

- I strongly suggest not mentioning the names of the sites that were included. This could lead to possible identification of participants, and on a worldwide scale, these names are not important.

**Response:** We accept the comment; we have omitted the name of the study sites.

- How were consents from minor participants given? To a guardian? What was the process?

**Response**: as we have indicated in the manuscript, for minors, both consent from their parents and assent from minors was obtained before data collection. We have invited parents of minors to the health facility to provide consent for their children.

Title: you briefly present gonorrhoeae findings… either take them out or include because they are not part of seroprevalence.

**Response**: as we have mentioned above we removed information related to gonorrhoeae from the manuscript.

Background:

The introduction/background section should be reorganized:

Response: We revised as suggested

- There is quite a bit of information that is not pertinent at this level of manuscript. Focus on: presenting the problem and solutions, the nature and scope of the problem investigated, a pertinent literature review to orient the reader, and the aims/objectives (https://guides.lib.uci.edu/c.php?g=334338&p=2249903)

**Response**: Baaed on the comment we have revised the background

In general- why are adolescents and young adults, especially females at increased risk for STI acquisition (and which types of STIs—the epidemiology of each varies so widely that they should not be grouped like this), what is known in Ethiopia already and why is it important to look into adolescents as well, how do you plan to study this population and these infections (last part of last paragraph of the intro).

**Response:** Baaed on the comment we have revised the back ground

- Each paragraph should focus on one topic (example: paragraph 3 jumps from youth and stis to economic burden). Stick to one topic per paragraph.

**Response:** We accept the comment, we broke paragraph 3 in two and we tried to link STI among youth and economic burden as shown in the manuscript with track change.

- what are ´list income countries? (line 86)

**Response:** we accept the comment we corrected as ‘low income countries’

Methods

Participant inclusion should be better discussed. How many were approached, how many declined? How did you end up with the final sample. Consider a flowchart to show this.

**Response:** the excepted number of source population during the study period was 2102. We proportionally allocated the sample size to all (four) study sites. We used systematic random sampling technique to recruit study participants. Whenever the client was not willing to participate we take the next person. We have used single proportion formula to calculate sample size. In case of refusal we go for the next client.

The sample collection and lab procedures vary between too much detail and not enough. I.e. you don´t need to go into the details of how to read a immunochromatic test strip but antimicrobial susceptibility testing should be better described.

**Response:** We accept the comment; we have omitted the details of the lab. We have omitted information related to Gonorrhoea.

- Why was RPR only used? And not (as WHO suggests) a RPR then antibody test (rapid test)?

**Response:** We did both as we have mentioned in the manuscript with a track change.

- What dilutions were carried out for RPR among all participants?

**Response:** We did not perform dilution

-Line 170 describes yet again the swabs? Only describe in one part of this section.

**Response:** we have omitted information related to NG

- Line 171 ´urogenital infection´ can mean different things to different people. What were the criteria and who made the call as to whether to include this testing or not?

**Response:** we have omitted information related to NG

- Line 188 who did consistency testing

**Response:** the consistency was checked by the principal investigator

-Line 189 are ´trained health professionals´ part of the author list? Indicate whom please

**Response:** No, they were not part of the authors list they are recruited for data collection.

-Line 217 and 218: cut point for what?

**Response:** it is a cut point for determination of statistically significant association as modified in the manuscript with track change.

- Check if operational definitions should be in this manuscript…if so, this seems like a very incomplete list. Please use a variable list that is complete and describes the measures correctly. Why only include heterosexual intercourse as transactional sex--This seems biased?

The variable list in the tables of the results is vague and needs better explanation.

**Response:** we accept the comment we included in the limitation section, we have also revised naming of variables listed in tables.

Result:

Results:

- age should be presented as a medium with IQR, (not mean with SD)

**Response:** we have corrected it.

- please do not repeat results from the tables in writeup of results.

**Response**: As recommended we have omitted some information presented in the text.

- Tables: Present tables 1 and 2 segregated by sex with p-values to show differences in demographics and behaviours.

**Response:** we have segregated table 2 and 3 (previous table 2 now it becomes two tables as you suggested).

-Group tables by knowledge/beliefs and behaivours. As Table 2 is now, it seems to jump around and is very hard to follow.

**Response:** based on the comment we have segregated them as table 2 for knowledge and table 3 fro behaviours and others.

- there needs to be a sex-segregated table for STI prevalence with p-values.

**Response:** in previous table 3 (now it is table four) in row 3 we have indicated distribution of prevalence of STI among males (6.3%) and females (13.9%). If we segregate other variables by sex it will complicate the table and also it is not the objective of our study.

- please report RPR findings by titer.

**Response:** we did not perform RPR titer

- Please report co-infections of STI- this is more important even than the % of STI by

Response: we have included confection under heading seroprevalence of STI

- Sti prevalence should be presented first by STI then as grouped STI (not grouped STI first)

**Response:** we have corrected it accordingly.

- Please use proper description of odds ratios: https://www.ncbi.nlm.nih.gov/pmc/articles/PMC2938757/ OR are not ´time higher than´ but rather : OR=1 Exposure does not affect odds of outcome, OR>1 Exposure associated with higher odds of outcome, OR<1 Exposure associated with lower odds of outcome´

**Response:** We have corrected it accordingly as shown in the manuscript with track change.

Discussion:

- This section should be reorganized. https://www.jclinepi.com/article/S0895-4356(13)00193-5/fulltext please follow in order to have a better organization of each paragraph. Again, please only include one topic per paragraph after paragraph 1. Example: Paragraph 1 should be an overview of major findings and results, etc.

Response: We have revised the discussion as shown in the manuscript with track change.

- Comparing chlamydia, gonorrhea and RPR findings to other studies that use distinct methods may not be accurate. Please check if PCR was not used for CT/NG on papers you cite, as well as rapid testing+RPR results. Make sure methods are similar.

**Response:** We accept the comment we have removed a study on gonorrhoea and chlamydia. Wherever it is applicable we have mentioned difference in laboratory methods for discrepancy.

- try not to compare with countries and studies in other regions that may not be comparable (i.e. focus on low and middle income countries- there are some published papers that are similar in Latin America- you can compare from there more accurately than the US.

**Response:** We accept the comment we omitted information from USA.

- behaviour outcomes and sti associations need to be discussion separately from knowledge/belief outcomes and sti associations as these phenomena and interventions are different from behavioural phenomena and interventions.

**Response:** In the study we have identified 3 factors which are significantly associated. We have tried to segregate them as suggest.

-There needs to be a clear policy and programme section to indicate how to better the sti outcomes in this population. I.e. HBsAg was very high—let´s focus on vaccination and followup studies on vaccination status to understand what populations are not being vaccinated.

Response: we agree with the comment accordingly we have include ‘Policy maker should find the reason for high prevalence of HBsAg and consider prevention strategies which may include vaccination and health education’

Limitations:

There are quite a few limitations to this study, which were not at all included in a section at the end of the discussion. For example:

- you´ve included facility-attending youth. These are not any youth, and they most likely have received greater sexuality education or have greater access to healthcare in the past. This needs to be discussed.

- interview face to face questionnaire biases responses of participants. Please discuss how.

- discuss all other limitations fully.

**Response:** We have included the limitations of the study after the discussion section.

6. PLOS authors have the option to publish the peer review history of their article (what does this mean?). If published, this will include your full peer review and any attached files.

If you choose “no”, your identity will remain anonymous but your review may still be made public.

Do you want your identity to be public for this peer review? For information about this choice, including consent withdrawal, please see our Privacy Policy.

Reviewer #1: No

Reviewer #2: No

[NOTE: If reviewer comments were submitted as an attachment file, they will be attached to this email and accessible via the submission site. Please log into your account, locate the manuscript record, and check for the action link "View Attachments". If this link does not appear, there are no attachment files.]

While revising your submission, please upload your figure files to the Preflight Analysis and Conversion Engine (PACE) digital diagnostic tool, https://pacev2.apexcovantage.com/. PACE helps ensure that figures meet PLOS requirements. To use PACE, you must first register as a user. Registration is free. Then, login and navigate to the UPLOAD tab, where you will find detailed instructions on how to use the tool. If you encounter any issues or have any questions when using PACE, please email PLOS at figures@plos.org. Please note that Supporting Information files do not need this step.
