# Supplementary material for: Sero-prevalence and associated factors of sexually transmitted infections among youth-friendly services Attendees
Source: PLoS One. 2023 Jan 23;18(1):e0279900. doi: 10.1371/journal.pone.0279900 (PMC9870153; doi:10.1371/journal.pone.0279900)
Supplement: Attachment — Submitted filename: Response to reviewers comment.docx [file pone.0279900.s003.docx]

PONE-D-22-12316R1

Sero-prevalence and associated factors of sexually transmitted infections among youth-friendly services attendants

PLOS ONE

Dear Dr. Ali,

Thank you for submitting your manuscript to PLOS ONE. After careful consideration, we feel that it has merit but does not fully meet PLOS ONE’s publication criteria as it currently stands. Therefore, we invite you to submit a revised version of the manuscript that addresses the points raised during the review process.

Please submit your revised manuscript by Dec 17 2022 11:59PM. If you will need more time than this to complete your revisions, please reply to this message or contact the journal office at [plosone@plos.org](mailto:plosone@plos.org). When you're ready to submit your revision, log on to https://www.editorialmanager.com/pone/ and select the 'Submissions Needing Revision' folder to locate your manuscript file.

Please include the following items when submitting your revised manuscript:

- A rebuttal letter that responds to each point raised by the academic editor and reviewer(s). You should upload this letter as a separate file labeled 'Response to Reviewers'.
- A marked-up copy of your manuscript that highlights changes made to the original version. You should upload this as a separate file labeled 'Revised Manuscript with Track Changes'.
- An unmarked version of your revised paper without tracked changes. You should upload this as a separate file labeled 'Manuscript'.

If you would like to make changes to your financial disclosure, please include your updated statement in your cover letter. Guidelines for resubmitting your figure files are available below the reviewer comments at the end of this letter.

If applicable, we recommend that you deposit your laboratory protocols in protocols.io to enhance the reproducibility of your results. Protocols.io assigns your protocol its own identifier (DOI) so that it can be cited independently in the future. For instructions see: <https://journals.plos.org/plosone/s/submission-guidelines#loc-laboratory-protocols>. Additionally, PLOS ONE offers an option for publishing peer-reviewed Lab Protocol articles, which describe protocols hosted on protocols.io. Read more information on sharing protocols at <https://plos.org/protocols?utm_medium=editorial-email&utm_source=authorletters&utm_campaign=protocols>.

We look forward to receiving your revised manuscript.

Kind regards,

Chuanyi Ning, Ph.D.
Academic Editor
PLOS ONE

[Note: HTML markup is below. Please do not edit.]

Reviewers' comments:

Reviewer's Responses to Questions

**Comments to the Author**

1. If the authors have adequately addressed your comments raised in a previous round of review and you feel that this manuscript is now acceptable for publication, you may indicate that here to bypass the “Comments to the Author” section, enter your conflict of interest statement in the “Confidential to Editor” section, and submit your "Accept" recommendation.

Reviewer #1: All comments have been addressed

Reviewer #2: All comments have been addressed

2. Is the manuscript technically sound, and do the data support the conclusions?

The manuscript must describe a technically sound piece of scientific research with data that supports the conclusions. Experiments must have been conducted rigorously, with appropriate controls, replication, and sample sizes. The conclusions must be drawn appropriately based on the data presented.

Reviewer #1: Yes

Reviewer #2: Partly

3. Has the statistical analysis been performed appropriately and rigorously?

Reviewer #1: Yes

Reviewer #2: Yes

4. Have the authors made all data underlying the findings in their manuscript fully available?

The [PLOS Data policy](http://www.plosone.org/static/policies.action#sharing) requires authors to make all data underlying the findings described in their manuscript fully available without restriction, with rare exception (please refer to the Data Availability Statement in the manuscript PDF file). The data should be provided as part of the manuscript or its supporting information, or deposited to a public repository. For example, in addition to summary statistics, the data points behind means, medians and variance measures should be available. If there are restrictions on publicly sharing data—e.g. participant privacy or use of data from a third party—those must be specified.

Reviewer #1: Yes

Reviewer #2: No

5. Is the manuscript presented in an intelligible fashion and written in standard English?

PLOS ONE does not copyedit accepted manuscripts, so the language in submitted articles must be clear, correct, and unambiguous. Any typographical or grammatical errors should be corrected at revision, so please note any specific errors here.

Reviewer #1: Yes

Reviewer #2: No

6. Review Comments to the Author

Please use the space provided to explain your answers to the questions above. You may also include additional comments for the author, including concerns about dual publication, research ethics, or publication ethics. (Please upload your review as an attachment if it exceeds 20,000 characters)

Reviewer #1: Now your manuscript is very good.

The researchers have been addressed all comments accordingly.

Response: Thank you for acknowledging our revised manuscript

Reviewer #2: In general, this is better written and described than the first version. However the authors should be careful of English use, especially odd capitalizations throughout.
It may be very helpful to find other studies published with Plos that are similar to see how the paragraph, especially the discussion is organized.

Response: Thank you for giving us additional comments. We have consulted other papers and corrected accordingly as shown in manuscript with track change.

Title Attendees vs attendants- check definitions

Response: Thank you for the comment, we have checked the definition we found ‘Attendees’ appropriate.

The section on knowledge and beliefs is big in the results section of the paper, but the title does not mention it- perhaps consider including.

Response: We feel this idea is included under associated factors; moreover if we include this information in the title the title will become lengthy.

Abstract Please check English throughout.

Response: We have revised.
Objective: to describe the seroprevalence of (which STIs)

Response: We have corrected as suggested.

Methods: do not capitalize syphilis, hepatitis B or hepatitis C. Define all acronyms you use in the abstract when you first mention them.

Response: We have corrected the capitalization and we defined the acronyms except the name of kits.

Results: please give individual STI results first, then grouped.

Response: We have presented like this based on based on the previous comment we also would like if stands as it is. Previous comment “Sti prevalence should be presented first by STI then as grouped STI (not grouped STI first)”

Consider using confidence interval vs confidence level

Response: We have used confidence interval (CI)

Give % and in (n/N). Don´t start a sentence with a number. What does the female AOR mean? Only use OR to two spots after the decimal. What is adjustment for? The section on OR needs to be rewritten as previously suggested to correctly report OR and AOR.

Response: We have corrected as suggested. The adjustment was for confounders.

Conclusions: comparable to other studies where and with what population?

Response: We have modified as shown in the manuscript with track change.

You still need to rewrite association results more clearly. STIs were associated with x not x with STI. Use terms more descriptive than ´High numbers´--- and there is an ´association with sexual activity without cndoms? What does the last line mean?

Response: We have corrected as suggested.

Background You describe the effects of STIs that you do not include (i.e.ectopic pregnancy, infertility, pelvic pain, inflammatory). Focus this on what you are studying only.

Response: We have omitted the information as recommended

Syphilis _may_ cause but doesn´t always or even usually cause neurological, cardiovascular or derm disease, same with neonatal effects).

Response: Yes we agree with the comment. We modified as ‘syphilis may cause…’

Broad statements such as STI prevalence ranges from 9-21% don’t say a whole lot. Focus on the STIs youstudy only, not all STIs in broad statements.

Response: We have omitted the statement as recommended.

Consider low and middle income country vs developing country.

Response: Thank you for the comment. We think we have provided sufficient background for the current study based on the previous comment.

The STIs you study don´t significantly affect reproductive potential (line 87).

Response: This is not our study; the information was taken from reference # 19 as a background. From our study syphilis may affect reproductive potential.

The scientific community prefers to use risk reduction rather than abstinence focuse (line 91-93)

Response: We think that starting sexual intercourse at early age without enough knowledge, attitude, and practice about STIs is also a risk factor especially in developing countries which should deserve a due attention.

Please give more background on Ethiopia and Hawassa in the introduction (not in methods)

Response: We moved some information from the method to background section as suggested.


Methods Please give types of HIV tests (antibody vs antigen? Lines 151+

Response: We have corrected accordingly.

Data analysis: what % of data were rechecked for correctness?

Response: 5% (if the question refers to pre-test). All quality control was run during data collection.

only define acronyms at first use from introduction onwards.

Response: We did as suggested

Consent: In minors: who gave consent before data collection? Minors or guardians?
Response: Consent was obtained from parents/guardians whereas assent was obtained from minors as shown in the manuscript with track change.

Results Did all participants answer all qustions? If not, n/N needs to be given for each variable.
% can be given before n (and put n in paranthases)

Response: The calculated sample size was 422; however, 416 (98.6%) were responded. The data presented in the result section reflect 416 participants. Every result is computed out of 416 respondents we have also presented the result as % (n/N) as shown in the manuscript with track change.

For the tables: consider not presenting in question format, but instead as reported HIV test, Has chewed khat, has smoked cigarettes, etc.

Response: We have corrected as suggested

Table 1: the variable monthly income is household or individual?
Response: it is monthly income of house hold as indicated in the manuscript with track change.

Table 2: what does the p-value indicate? Difference between sexes? Include in the table title what it means.

Response: It was included based on the previous comment. Now, we have included in the title “…..among males and females…”

Again, make sure the acronyms for STIs have been presented previously.

Response: We have defined STIs in the introduction section.

Factors associated with STIs: no need to reexplain methods.

Response: We omitted the subheading and modified the other subheading as ‘Seroprevalence of STI and its predictors’

´Grouped STs were associated with x, y and z or x, y and z were associated with increased odds of grouped STI – and make sure you´ve defined what grouped STIs are in methods)

Response: We have defined STI under operational definition

Discussion Have a look again at what to include in the first paragraph of a discussion- you can include some overall riskfactors as well as STI results.

Response: We have included factors associated with STI in the first paragraph

Lines starting 250: are these studies with reports of STI? Or findings in laboratory testing? It seems like as It is written that participants of these studies had to self-report their sti diagnosis (which is never going to be accurate)
Response: they are not self-report they are the findings of studies (reference)

Paragraph 3 needs to be restructured. Keep to one topic. The highest prevalence is HBV- what does that mean for vaccination? Further research should look at vaccination status.

Response: We think the reviewer is referring to paragraph 4. We presented them together because of their similarity in route of transmission and pathogenesis (HBV, HCV).

All the paragraphs with comparisons from other LMIC can be grouped into one paragraph.

Response: Thank you for the comment. There are different ways of organizing paragraphs; we preferred to organize the discussion based on our objectives and nature of STIs.

Make sure your study is comparable to what you present. What is given from Rhode Island doesn´t make sense (in incarcerated populations)

Response: We agree with the comment, we omitted it.

Make sure your comparisons make sense. Your syphilis prevalence of 0.7% does to compare with Brazilian prevalence.

Response: We have corrected it as “….is low compared to….”

You should have a section on ´policy and program recommendations´ or at the very least be mentioning this throughout the discussion. A whole section would be better though.

Response: We have included heading ‘policy and program recommendation’

Limitations: use proper limitations and bias vocabulary <https://jech.bmj.com/content/58/8/635>

Response: We have modified the limitation somehow after refereeing to the link provided (Thank you)

Conclusions: you need to include program recommendations.

Response: we have included.

Please define abbreviations within text as well (when you first use them)

Response: we dis as suggested.

7. PLOS authors have the option to publish the peer review history of their article ([what does this mean?](https://journals.plos.org/plosone/s/editorial-and-peer-review-process#loc-peer-review-history)). If published, this will include your full peer review and any attached files.


If you choose “no”, your identity will remain anonymous but your review may still be made public.


**Do you want your identity to be public for this peer review?** For information about this choice, including consent withdrawal, please see our [Privacy Policy](https://www.plos.org/privacy-policy).

Reviewer #1: No

Reviewer #2: No

[NOTE: If reviewer comments were submitted as an attachment file, they will be attached to this email and accessible via the submission site. Please log into your account, locate the manuscript record, and check for the action link "View Attachments". If this link does not appear, there are no attachment files.]

While revising your submission, please upload your figure files to the Preflight Analysis and Conversion Engine (PACE) digital diagnostic tool, <https://pacev2.apexcovantage.com/>. PACE helps ensure that figures meet PLOS requirements. To use PACE, you must first register as a user. Registration is free. Then, login and navigate to the UPLOAD tab, where you will find detailed instructions on how to use the tool. If you encounter any issues or have any questions when using PACE, please email PLOS at [figures@plos.org](mailto:figures@plos.org). Please note that Supporting Information files do not need this step.
